# Rutin Alleviates Oxidative Stress in Chicken Hepatocytes by Inhibiting Ribotoxic Stress Response

**DOI:** 10.3390/ani16111654

**Published:** 2026-05-28

**Authors:** Cangning Zhang, Tao Li, Chengfeng Zhao, Liqin Zhao, Duniesky Rodriguez Acosta, Osmany Chacon Chacon, Liumei Sun, Weiguo Zhao, Liang Qu, Manman Shen

**Affiliations:** 1 Jiangsu Key Laboratory of Sericultural and Animal Biotechnology, School of Biotechnology, Jiangsu University of Science and Technology, Zhenjiang 212100, China; zhangcangning1999@163.com (C.Z.); taoli912@126.com (T.L.); inmitlessness@163.com (C.Z.); 18717651933@163.com (L.Z.); sun.liumei@163.com (L.S.); wgzsri@126.com (W.Z.); 2 Key Laboratory of Silkworm and Mulberry Genetic Improvement, Ministry of Agriculture and Rural Affairs, Sericultural Scientific Research Center, Chinese Academy of Agricultural Sciences, Zhenjiang 212100, China; 3 Protein and Bionatural Products Research Center, Ministry of Agriculture, Havana 999075, Cuba; duniesky838@gmail.com (D.R.A.); osmanichacon75@gmail.com (O.C.C.); 4 Jiangsu Institute of Poultry Science, Yangzhou 225125, China; liangquyz@126.com

**Keywords:** poultry, liver, rutin, oxidative stress, ribotoxic stress response

## Abstract

Oxidative stress (OS) significantly impairs poultry production; however, the protective mechanisms of rutin in the chicken liver remain unclear. In this study, we investigated the regulatory effects of rutin on oxidative stress and lipid metabolism using H_2_O_2_-exposed primary hepatocytes derived from chicken embryos. Specifically, we focused on antioxidant indicators, the Nrf2/Keap1 signaling pathway, and the ZAKα-mediated ribotoxic stress response (RSR). Results showed that pretreatment with 800 μmol/L rutin significantly alleviated oxidative damage by increasing superoxide dismutase (SOD) and total antioxidant capacity (T-AOC) activities, while decreasing malondialdehyde (MDA) and reactive oxygen species (ROS) levels. This protective effect was mediated by activation of the Nrf2/Keap1 signaling pathway and its downstream effectors, HO-1 and SOD2. Concurrently, rutin suppressed lipid accumulation, as evidenced by reduced levels of triglyceride (TG), total cholesterol (T-CHO), and low-density lipoprotein cholesterol (LDL-C), along with the downregulation of SREBP1 expression. Mechanistically, we found that rutin inhibited ZAKα and its downstream signaling, the p38/JNK pathway, which is associated with RSR. Crucially, co-treatment with ZAKα overexpression significantly attenuated the protective effects of rutin on both antioxidant defense and lipid regulation, confirming that rutin functions by inhibiting ZAKα-mediated signaling to restore Nrf2 activity. Molecular docking revealed a binding energy of −8.6 kcal/mol between rutin and ZAKα, verifying that ZAKα serves as a potential target for rutin. Collectively, these findings elucidate a novel mechanism whereby rutin enhances antioxidant defense and inhibits lipid deposition via the ZAKα-p38/JNK-Nrf2 signaling pathway, providing a theoretical basis for the application of rutin as a targeted regulator in poultry health.

## Simple Summary

Oxidative stress in the liver is a major challenge in poultry production, often leading to compromised health and economic losses. This study investigated the ability of rutin, a natural plant compound, to protect chicken hepatocytes against oxidative damage. Using hepatocytes exposed to H_2_O_2_ to simulate physiological stress, we found that rutin pretreatment significantly enhanced the cells’ antioxidant defenses and reduced harmful lipid accumulation. We discovered that rutin achieves this by inhibiting a specific stress-response protein called ZAKα. When we overexpressed ZAKα in the hepatocytes, rutin’s protective effects were notably weakened. These results indicate that rutin effectively protects chicken hepatocytes by targeting ZAKα, suggesting it could be a valuable natural supplement to improve liver health and overall productivity in poultry flocks.

## 1. Introduction

Poultry farming, as a vital component of global livestock production, faces significant challenges due to environmental changes and management practices [1]. Factors such as high-density housing and nutritional imbalances trigger stress responses that impair growth performance, feed conversion ratios, production performance, and overall economic viability [2,3,4]. Central to these challenges is oxidative stress (OS), a critical physiological bottleneck that disrupts metabolic homeostasis and deteriorates carcass quality through systemic lipid peroxidation and the depletion of antioxidant defenses [5,6]. OS occurs when the generation of reactive oxygen species (ROS) exceeds the scavenging capacity of endogenous antioxidant systems [7]. The avian liver, characterized by intense lipid turnover, is particularly vulnerable to oxidative damage, leading to excessive ROS production and impairing the antioxidant defense system [8,9]. Under stress conditions, ROS production is upregulated, while the activity of antioxidant enzymes, including superoxide dismutase (SOD), catalase (CAT), and heme oxygenase-1 (HO-1), is suppressed [10,11]. This failure in radical scavenging triggers a marked increase in lipid peroxidation, as evidenced by elevated levels of malondialdehyde (MDA) [12]. Simultaneously, the imbalance between oxidation and antioxidant defense inhibits growth performance and increases mortality [13]. Given that oxidative stress is a fundamental driver of hepatic pathogenesis, the Nuclear Factor Erythroid 2-Related Factor 2(Nrf2)/Kelch-like ECH-associated protein 1 (Keap1) pathway has emerged as a critical cytoprotective mechanism designed to restore redox homeostasis [14]. Therefore, nutritional modulation of Nrf2-driven antioxidant responses in the liver offers an approach to counteract oxidative damage in poultry.

To alleviate oxidative stress in poultry, dietary inclusion of natural flavonoids has emerged as a promising strategy due to their diverse biological activities [15,16]. Rutin (5,7,3′,4′-tetrahydroxy-3-rutinoside), a bioactive glycoside also known as Vitamin P, is widely found in various vegetables and fruits [17,18]. Rutin is recognized for its multiple pharmacological effects, including antioxidant, free-radical scavenging, cardioprotective, antiviral, and anti-inflammatory properties, and it plays a key role in modulating glucose and lipid homeostasis with favorable absorption both in vivo and in vitro [19]. In poultry, rutin acts as a potent antioxidant, posing anti-inflammatory and lipid-regulating properties, improving intestinal morphology and microbiota structure [17,20]. Notably, it has been shown to prevent hepatic necrosis in chickens by modulating mitogen-activated protein kinase (MAPK) signaling pathways and elevating total antioxidant capacity (T-AOC) [21]. Although evidence from rodent models indicates that rutin effectively reduces triglycerides (TG) and total cholesterol (T-CHO) [22], its precise regulatory role in oxidative stress and lipid metabolism in the chicken liver remains largely unexplored. This knowledge gap is particularly significant given that the avian liver serves as the primary site for de novo lipogenesis and cholesterol homeostasis, playing a crucial role in post-hatch growth and health [23,24,25].

MAPKs, particularly p38 and c-Jun N-terminal protein kinase (JNK), are canonical effectors in the cellular response to oxidative stress, with their phosphorylation regulating cell fate and inflammatory responses [26]. However, most previous studies have focused primarily on p38 and JNK as downstream responders, often neglecting the upstream molecular sensors that link oxidative insults to these kinase cascades. Recent mechanistic insights have identified MAP3K20 (ZAKα) as a ribosome-interacting protein and a key sensor of the ribotoxic stress response (RSR) [27]. Under oxidative injury, ZAKα detects translational stalling via its C-terminal domain and subsequently phosphorylates p38 and JNK, thereby converting ribosomal damage into biochemical stress signals [28,29,30]. Inhibition of ZAKα has been shown to block the p38-mediated dysregulation in myeloid differentiation [31]. Therefore, investigating ZAKα-driven activation of downstream p38/JNK signaling is essential to elucidate the specific regulatory effects of oxidative damage. However, the role of the ZAKα-p38/JNK axis remains undefined in the avian liver.

In light of the persistent threat of OS to poultry health, this study utilizes chicken embryonic primary hepatocytes to evaluate the effects of rutin on oxidative stress. We aim to evaluate the protective effects of rutin against OS and lipid metabolism dysregulation in hepatocytes. Specifically, our objective is to elucidate whether rutin mitigates oxidative damage and hepatic lipid deposition by targeting the ZAKα-mediated ribotoxic stress response and its downstream p38/JNK signaling pathway. By investigating this previously unexplored pathway in the avian liver, our study seeks to uncover a novel molecular mechanism of rutin and provide a theoretical foundation for its application as a nutritional regulator in poultry health.

## 2. Materials and Methods

### 2.1. Isolation and Culture of Chicken Primary Hepatocytes

Fertilized eggs were obtained from the Jiangsu Institute of Poultry Science and incubated at 37.8 °C with 60% relative humidity following standard protocols. Primary hepatocytes were isolated from 14-day-old embryos that were humanely euthanized by decapitation, following procedures previously described by our laboratory [32,33]. Briefly, the embryonic liver was collected and cut into approximately 1 mm^3^ pieces. The minced liver tissues were washed with PBS three times to remove blood cells. The tissues were then digested with trypsin for 15 min at 37 °C, and the digestion was terminated using complete medium. The mixture was filtered sequentially through 100 μm, 70 μm, and 40 μm meshes. The isolated hepatocytes were cultured in DMEM medium supplemented with 10% fetal bovine serum (FBS, BioChannel, Nanjing, China), 1% penicillin-streptomycin, and 5 μg/mL transferrin. Cells were cultured at 37 °C in a 5% CO_2_ incubator. Before seeding, culture plates were coated with 0.1% gelatin for 1 h, washed twice with PBS, and then cells were seeded. All embryo-related procedures were approved by the Animal Care and Use Committee of Jiangsu University of Science and Technology (GJ2024SJ05, Zhenjiang, China).

### 2.2. Isolation and Identification of Primary Hepatocytes from Chicken Embryos

Cells were seeded in 12-well plates. After the hepatocytes had completely adhered and were in optimal growth conditions, glycogen periodic acid–Schiff (PAS) staining was performed to identify the hepatocytes. The procedure was as follows: the cells were fixed with PAS fixative for 10–15 min, then washed with water and air-dried. An oxidizing reagent was added, and the cells were incubated at room temperature for 15–20 min, followed by two washes with distilled water. Schiff’s staining solution was then added, and the cells were stained in the dark at room temperature for 10–20 min, followed by rinsing under water for 5 min. Mayer’s hematoxylin staining solution was added for counterstaining for 1–2 min, after which the cells were washed with distilled water, air-dried, and observed under a microscope (Olympus, Tokyo, Japan).

### 2.3. Establishment of Oxidative Stress Model and Rutin Treatment

Cells were seeded into 96-well plates and cultured for 24 h to reach confluence. To establish the oxidative stress model, cells were treated with H_2_O_2_ at the gradient concentrations (400, 600, 800, 1000, 2000, 3000, 4000, 5000, and 6000 μmol/L; Cas: 7722-84-1; Longda, Zhenjiang, China) for 3 h. Cell viability was detected using the Cell Counting Kit-8 (CCK-8, APExBIO, Shanghai, China). Briefly, 10 μL of CCK-8 solution was added to each well, and the cells were incubated for 2 h. Absorbance was measured at 450 nm using a microplate reader (BioTek, Winooski, Vermont, USA). Based on the cell viability data, the optimal H_2_O_2_ concentration was selected for subsequent experiments. To screen for the optimal concentrations of rutin (Purity ≥98%; B20771, Yuanye, Shanghai, China), according to previously reported concentrations of rutin [34,35], hepatocytes were pre-treated with various concentrations of rutin (400, 600, 800, 1000, 1500, 2000, 2500, and 3000 μmol/L) for 24 h before H_2_O_2_ exposure. Cell viability was detected as described above to determine the optimal concentration of rutin.

### 2.4. Detection of Antioxidant Indicators and Lipid Metabolism

After treatment, cell supernatants were collected to determine the levels of MDA (A003-1-1), SOD (A001-1-2), CAT (A0071-1-1), T-AOC (A015-1-1), as well as the lipid profiles of TG (A110-1-1), T-CHO (A111-1-1), high-density lipoprotein cholesterol (HDL-C, A112-1-1), and LDL-C (A113-1-1). These measurements were performed using commercial kits from Jianchen Company (Nanjing, China), strictly following the manufacturer’s instructions.

### 2.5. Measurement of Intracellular ROS Levels

Intracellular ROS levels were determined using the fluorescent probe DCFH-DA (S0033S, Beyotime, Shanghai, China). Briefly, treated cells were washed with PBS and incubated with 10 μM DCFH-DA at 37 °C for 30 min in the dark. After washing twice with PBS to remove extracellular dye, fluorescence emission was captured using an inverted fluorescence microscope (Olympus, Tokyo, Japan; excitation wavelength: 488 nm; emission wavelength: 525 nm).

### 2.6. Construction of the ZAKα Overexpression Vector

The coding sequence (CDS) of the chicken ZAKα gene (GeneBank Accession No. XM_421996.8) was amplified from chicken liver cDNA using primers containing EcoRI and XhoI restriction sites (F: ccggaattcCATGGAGATGGACGGGGATG R: ccgctcgagGCTCGAACGTCGCTCTCATA). The amplified PCR product was purified, digested, and cloned into the pcDNA3.1 vector to generate the ZAKα overexpression vector (OE-ZAKα). Sequence fidelity was confirmed by Sanger sequencing.

### 2.7. Western Blot Analysis

After treatment, total protein was extracted from hepatocytes using RIPA lysis buffer containing 1% PMSF. Lysates were centrifuged at 12,000 rpm for 5 min, and the total protein content was determined using a BCA (A045-4-2) protein assay kit (Jiancheng, Nanjing, China). Equal amounts of protein were separated by SDS-PAGE and transferred into PVDF membranes, which were then blocked with 5% nonfat milk for 1 h at room temperature. The membranes were incubated overnight with the following primary antibodies: Nrf2 (WL02135), Keap1 (WL03285), HO-1 (WL02400), and SOD2 (WL02506) from Wanleibio (Beijing, China); Sterol Regulatory Element-Binding Protein 1 (SREBP1) (bs-1402R), p-p38 (bs-2210R), and p38 (bs-28027R) from Bioss (Beijing, China); ZAKα (28761-1-AP) from Proteintech (Wuhan, China); GAPDH (M2006F) from Abmart (Shanghai, China); p-JNK (ABP50351) and JNK (ABP51663) from Abbkine (Wuhan, China); Tubulin-β (AP0064) from Bioworld (Nanjing, China), and β-actin (ZB15001-HRP) from Servicebio (Wuhan, China). Following three washes with TBST, membranes were incubated with HRP-conjugated secondary antibodies for 1 h at room temperature. Protein bands were visualized using an ECL kit and quantified using ImageJ software v2.0 (National Institutes of Health, USA). GAPDH, Tubulin-β, or β-actin served as loading controls.

### 2.8. Molecular Docking of Rutin with ZAKα

The PDB structure of ZAKα was predicted using AlphaFold, and the three-dimensional structure of rutin was obtained from the PubChem database. Water molecules and small-molecule ligands within the protein were removed using PyMOL software v1.7.6. Ligand and receptor preparation were performed with AutoDock Tools v. 1.5.6. Computational docking simulations of the protein–rutin complex were carried out using AutoDock Vina, and structural visualization was achieved with PyMOL.

### 2.9. Statistical Analysis

Each experiment was performed with at least three biological replicates. All data are presented as mean ± standard error of mean (S.E.M). Statistical analyses were performed using SPSS statistical software v 22 (SPSS Inc., Chicago, IL, USA). Statistical differences between groups were assessed using one-way analysis of variance (ANOVA), followed by the Least Significant Difference (LSD). In contrast, differences within each group were tested using Student’s t-test. *p* < 0.05 was considered statistically significant.

## 3. Results

### 3.1. Identification of Primary Hepatocytes from Chicken Embryos

**Figure 1:**
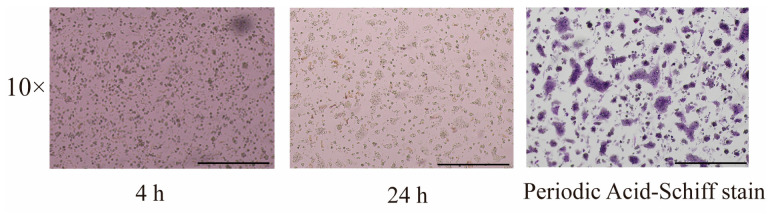
Identification of Primary Hepatocytes from Chicken Embryo, scale bar = 200 μm.

As shown in Figure 1, primary chicken embryo hepatocytes were successfully isolated and characterized. After 4 h of culture, most cells adhered to the substrate and appeared round or oval with distinct margins. After 24 h, cells extended filopodia and exhibited the typical spreading morphology of hepatocytes. Periodic acid-schiff (PAS) staining revealed a magenta-positive reaction for cytoplasmic glycogen, confirming hepatocyte identity.

### 3.2. Optimization of the Oxidative Stress Model and Optimal Rutin Concentration

**Figure 2:**
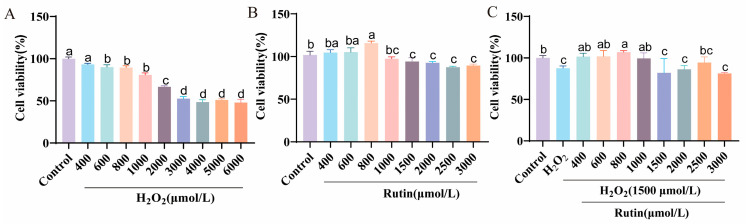
Screening for Optimal Concentrations of H_2_O_2_ and Rutin in Primary Chicken Embryo Hepatocytes. (**A**) Dose–response effect of H_2_O_2_ on hepatocyte viability after 3 h treatment; (**B**) Changes in cell viability following a 24 h pretreatment with various concentrations of rutin; (**C**) Recovery of cell viability after a 24 h pretreatment with different concentrations of rutin, followed by 3 h injury with 1500 μmol/L H_2_O_2_. Different letters indicate significant differences between groups (*p* < 0.05).

As shown in Figure 2A, treatment with H_2_O_2_ for 3 h induced cell damage, reducing cell viability to approximately 70–80% at concentrations of 1000–2000 μmol/L (*p* < 0.05). Notably, cytotoxicity plateaued at ~50% viability for concentrations exceeding 2000 μmol/L. Therefore, a treatment regime of 1500 μmol/L for 3 h was selected to establish the oxidative stress model. To screen the optimal cytoprotective concentration of rutin, cells were pre-treated with different concentrations of rutin before H_2_O_2_ exposure. Rutin pretreatment significantly increased cell viability, with maximal efficacy observed at 800 μmol/L under both basal conditions (Figure 2B) and in the presence of H_2_O_2_-induced oxidative stress (Figure 2C). This concentration was subsequently adopted for all further experiments.

### 3.3. Rutin Alleviates H_2_O_2_-Induced Oxidative Stress and Reduces Lipid Synthesis

**Figure 3:**
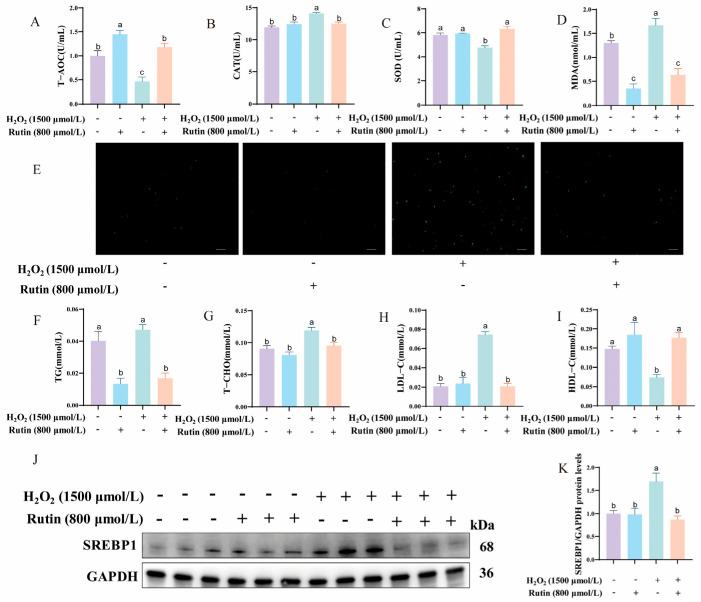
Regulatory Effects of Rutin on Antioxidant Capacity and Lipid Metabolism in H_2_O_2_-Induced Hepatocytes. (**A**–**D**) Levels of total antioxidant capacity (T-AOC), catalase (CAT), superoxide dismutase (SOD), and malondialdehyde (MDA); (**E**) Representative images and quantitative analysis of ROS fluorescence intensity, scale bar = 100 μm; (**F**–**I**) Levels of triglycerides (TG), total cholesterol (T-CHO), low-density lipoprotein cholesterol (LDL-C), and high-density lipoprotein cholesterol (HDL-C). (**J**,**K**) Representative Western blot bands and quantitative analysis of SREBP1 protein expression. Different letters indicate significant differences among groups (*p* < 0.05).

Since redox homeostasis is critical for hepatic function, we first evaluated the impact of rutin on antioxidant defense systems. Exposure to H_2_O_2_ caused severe oxidative imbalance, characterized by a marked reduction in SOD activity and T-AOC levels, accompanied by an elevation in CAT activity and MDA accumulation (*p* < 0.05, Figure 3A–D). Rutin pretreatment significantly attenuated H_2_O_2_-induced intracellular ROS generation (*p* < 0.05, Figure 3E). Concomitant with oxidative damage, H_2_O_2_ exposure enhanced lipid metabolism, leading to elevated levels of TG, T-CHO, and LDL-C, alongside a reduction in HDL-C (*p* < 0.05, Figure 3F–I). Western blot analysis indicated that this metabolic shift was associated with upregulation of SREBP1, a master regulator of de novo lipogenesis (*p* < 0.05, Figure 3J,K). Rutin pretreatment significantly downregulated SREBP1 expression and normalized the lipid profile (*p* < 0.05), indicating that rutin exerts dual protective effects by enhancing antioxidant defenses and suppressing SREBP1-mediated lipogenesis.

### 3.4. Rutin Modulates the Nrf2/Keap1 and ZAKa-p38/JNK Signaling Pathways

**Figure 4:**
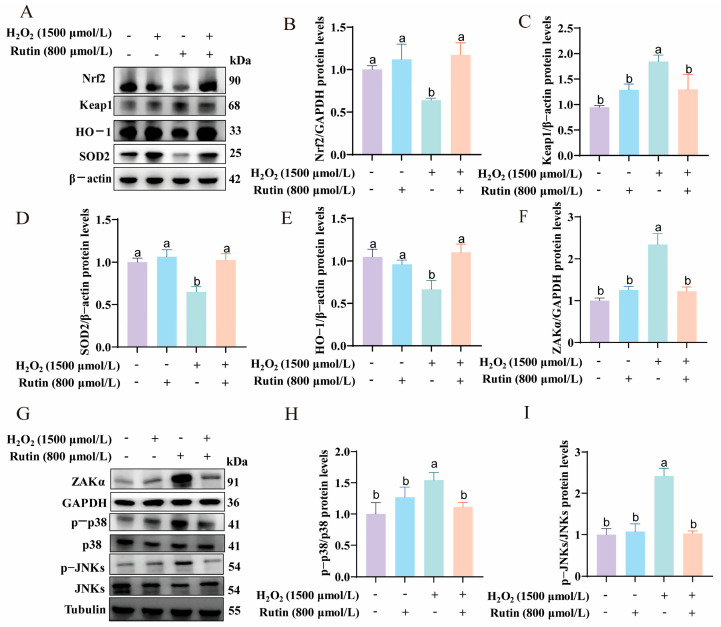
Effects of Rutin on the Nrf2/Keap1 Signaling Pathway and Ribosomal Stress Response. (**A**–**F**) Levels of Nrf2, Keap1, and their downstream HO-1 and SOD2; (**G**–**I**) Levels of the ZAKa-p38/JNK pathway. Different letters indicate significant differences among groups (*p* < 0.05).

To explore the potential mechanisms underlying rutin’s effects, we examined alterations in the Nrf2/Keap1 and ZAKa-p38/JNK signaling pathways. As illustrated in Figure 4, compared with the control group, H_2_O_2_ treatment significantly reduced the expression of Nrf2 and its downstream effectors, SOD2 and HO-1 (*p* < 0.05), while increasing the expression of Keap1, ZAKα, and the phosphorylation of p38 and JNKs (*p* < 0.05). Rutin pretreatment significantly alleviated these detrimental changes in the Nrf2/Keap1 signaling pathway (*p* < 0.05) and concurrently suppressed the activation of the ZAKa-p38/JNK pathway. These data demonstrate that rutin activates the Nrf2/Keap1 signaling pathway while inhibiting the ribosomal stress response.

### 3.5. Construction of the ZAKα Overexpression Vector and Analysis of Related Parameter Changes

**Figure 5:**
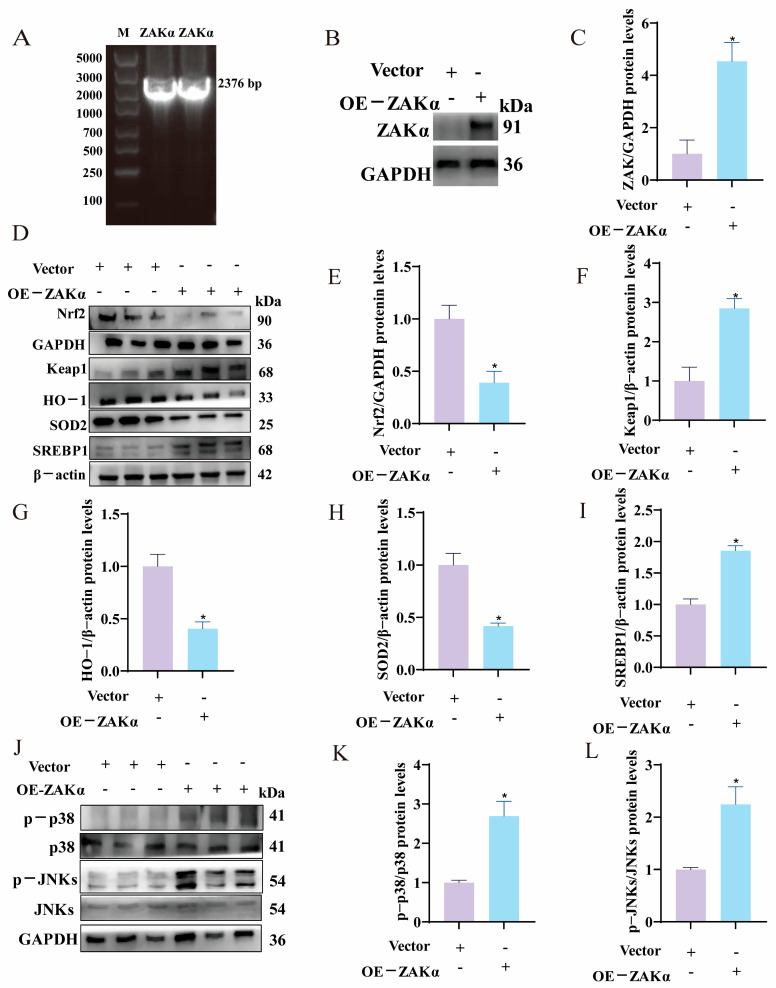
Construction of the ZAKα Overexpression Vector and Associated Changes in Related Parameters. (**A**) Agarose gel electrophoresis validation of the target region of the ZAKα CDS; (**B**,**C**) Validation of ZAKα overexpression efficiency at the protein level; (**D**–**I**) Protein expression of Nrf2/Keap1 signaling components (Nrf2, Keap1, HO-1, SOD2); (**I**) Western blot analysis of SREBP1 protein expression; (**J**–**L**) Western blot analysis of p-p38/p38 and p-JNKs/JNKs protein expression. * Means vs. Vector group, with * means *p* < 0.05.

To validate the upstream role of ZAKα, we constructed a ZAKα overexpression vector and confirmed its size at 2376 bp (Figure 5A), corresponding to GenBank accession XM_421996.8. Transfection efficiency was verified by a significant increase in ZAKα protein levels (*p* < 0.05, Figure 5B,C). To assess the effects of ZAKα overexpression on hepatocyte function, we measured relevant indicators. As shown in Figure 5D–L, compared with the empty vector control group, protein levels of Nrf2, HO-1, and SOD2 were significantly downregulated in the ZAKα overexpression group (*p* < 0.05), while levels of Keap1, SREBP-1, phosphorylated p38 (p-p38), and phosphorylated JNKs (p-JNKs) protein expression were significantly upregulated (*p* < 0.05).

### 3.6. Overexpression of ZAKα Attenuates the Inhibitory Effect of Rutin on the p-p38/p-JNK Signaling Pathway

**Figure 6:**
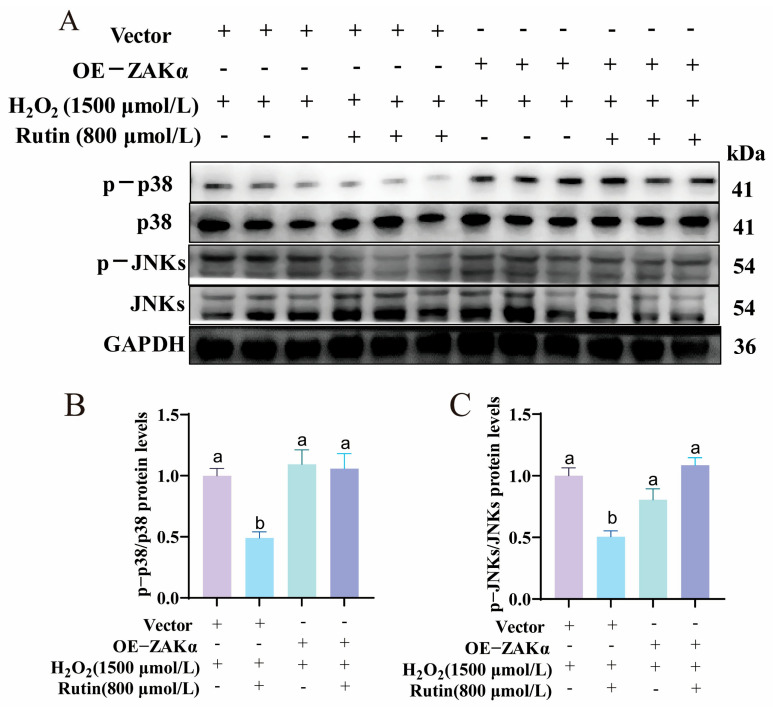
Overexpression of ZAKα Attenuates the Inhibitory Effect of Rutin on the p-p38/p-JNKs Signaling Pathway; (**A**–**C**) Effects of ZAKα overexpression on p-p38 and p-JNKs protein expression in H_2_O_2_-treated cells. Different letters indicate significant differences between groups (*p* < 0.05).

Assays demonstrated that rutin pretreatment significantly suppressed the phosphorylation of p38 and JNKs in the vector control groups (Figure 6A–C). However, this inhibitory effect was weakened in the OE-ZAKα treatment group. These results confirm that ZAKα overexpression enhances the ribotoxic stress response and attenuates rutin’s inhibitory effect on the ZAKα-p38/JNK pathway.

### 3.7. Overexpression of ZAKα Counteracts the Antioxidative Effects of Rutin

**Figure 7:**
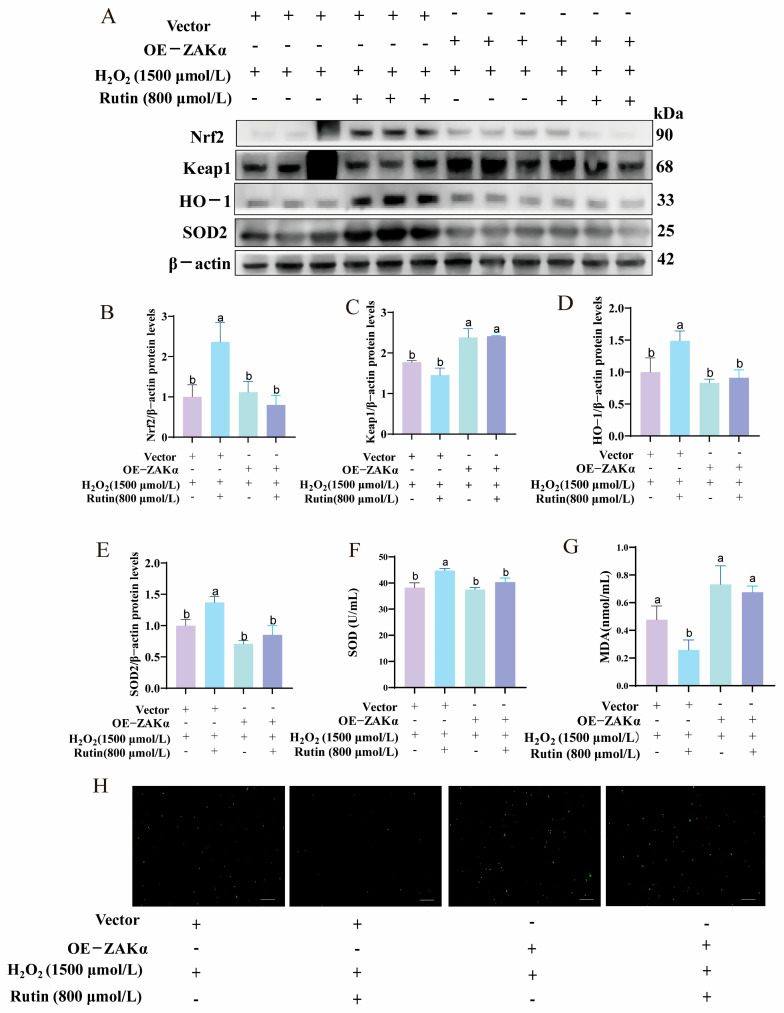
Overexpression of ZAKα Suppresses the Protective Effect of Rutin Against H_2_O_2_-induced Oxidative Stress. (**A**–**E**) Protein expression levels of Nrf2/Keap1 signaling pathways (Nrf2, Keap1, HO-1, SOD2); (**F**,**G**) Levels of superoxide dismutase (SOD) and malondialdehyde (MDA) across groups; (**H**) ROS fluorescence intensity across groups. Scale bar = 100 μm. Different letters indicate significant differences between groups (*p* < 0.05).

To investigate whether the antioxidant capacity of rutin relies on ZAKα inhibition, we examined the Nrf2/Keap1 signaling pathway, antioxidant markers, and intracellular ROS levels (Figure 7A–H). ZAKα overexpression counteracted the rutin-induced upregulation of Nrf2, HO-1, and SOD2 (*p* < 0.05), while reducing Keap1 expression (*p* < 0.05). Mechanistically, the protective effects of rutin—reflected by increased SOD activity and decreased MDA and ROS levels—were significantly diminished by ZAKα overexpression *(p* < 0.05). Cells co-treated with OE-ZAKα and rutin exhibited oxidative damage markers comparable to or exceeding those in the H_2_O_2_-only group. These results confirm that the Nrf2-mediated antioxidant response induced by rutin is mechanistically dependent on the suppression of ZAKα signaling.

### 3.8. Overexpression of ZAKα Attenuates the Lipid-Lowering Effects of Rutin

**Figure 8:**
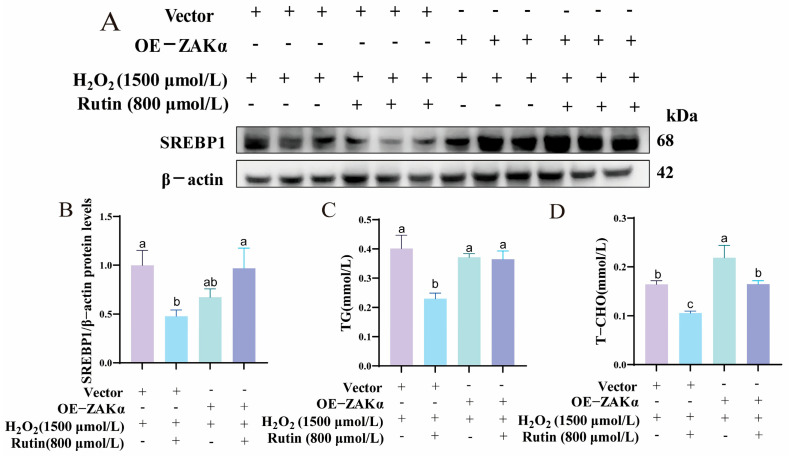
Overexpression of ZAKα Counteracts the Regulatory Effects on Lipid Metabolism in Hepatocytes. (**A**,**B**) Western blot analysis of SREBP1 protein expression. (**C**,**D**) Determination of triglyceride (TG) and total cholesterol (T-CHO) content. Different letters indicate significant differences between groups (*p* < 0.05).

The regulatory effects of ZAKα overexpression on lipid metabolism are shown in Figure 8A–D, demonstrating that rutin pre-treatment significantly reduced the accumulation of TG and T-CHO, and downregulated SREBP1 expression (*p* < 0.05). However, overexpression of ZAKα significantly diminished these beneficial effects, preventing the rutin-induced downregulation of SREBP1 and TG. Interestingly, despite ZAKα overexpression, rutin maintained its capacity to significantly reduce T-CHO levels (*p* < 0.05). These findings demonstrate that the ZAKα-mediated RSR pathway acts as a negative regulator of rutin’s beneficial effects, and that its inhibition is essential for rutin to exert its lipid-lowering and antioxidant effects.

### 3.9. Molecular Docking of ZAKα with Rutin

**Figure 9:**
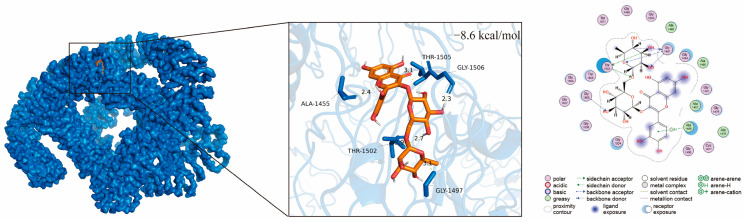
Schematic Diagram Illustrating the Molecular Docking Between Rutin and ZAKα.

The molecular docking and binding energy of ZAKα with rutin are shown in Figure 9. The binding energy was −8.6 kcal/mol, indicating a strong interaction between the two molecules. Hydrogen bond interactions were formed between rutin and the amino acid residues ALA-1455, THR-1502, THR-1505, GLY-1506, and GLY-1497 of the ZAKα protein, with distances of 2.4, 2.9, 3.1, 2.3, and 3.1, respectively. These hydrogen bonds play a critical role in stabilizing the binding between rutin and ZAKα.

## 4. Discussion

In the modern intensive poultry industry, the liver serves as a metabolic organ but is uniquely vulnerable to oxidative insults, which impair flock health and productivity [7]. Rutin, a flavonoid compound widely found in plant-based foods and medicinal materials, plays an important role in scavenging free radicals and exerting anti-inflammatory effects [36,37,38]. However, its hepatoprotective effects on the ribotoxic stress pathway have remained largely unexplored. Our study provides the first evidence that rutin orchestrates a dual-protective mechanism in avian hepatocytes, restoring redox homeostasis by potentiating the Nrf2/Keap1 axis and concurrently normalising lipid metabolism by suppressing SREBP1. Crucially, we identify the ribotoxic stress sensor ZAKα as a pivotal upstream effector, whose inhibition is mechanistically required for rutin to exert its full cytoprotective and lipid-lowering efficacy. Oxidative stress arises from an imbalance between the generation and scavenging of ROS, accompanied by elevated H_2_O_2_ levels [39]. Establishing a precise H_2_O_2_-induced injury model is essential for evaluating potential antioxidant interventions. Our data confirm that H_2_O_2_ exposure disrupts the hepatic antioxidant machinery. Consistent with previous protocols established in the avian chicken leghorn male hepatoma cell line (LMH) cells [10] and the human hepatoblastoma cell line (HepG2) [40], where the corresponding concentrations of H_2_O_2_ were 1000 μmol/L and 600 μmol/L, respectively, we utilized cell viability as the evaluation metric to calibrate the stress threshold. In this study, 1000 µmol/L H_2_O_2_ induced only mild damage, whereas 2000 µmol/L caused severe damage. Based on the cell viability data, a concentration of 1500 µmol/L H_2_O_2_ was selected for subsequent experiments to establish a model of moderate oxidative stress. Using this validated model, we demonstrate that rutin exerts a potent protective effect, reinforcing its role as a highly efficient free radical scavenger in avian hepatocytes. While rutin is conventionally applied at doses below 200 μmol/L, we employed a concentration of 800 μmol/L, based on a previous report [34] and our own experimental validation, which identified this dose as the optimal balance between safety and viability in chicken hepatocytes. This discrepancy underscores cell type-specific responses to rutin, necessitating tailored dose optimization across different experimental models.

Mechanistically, rutin’s protective effects depend on the modulation of the Nrf2/Keap1 signaling pathway. Under normal physiological conditions, Nrf2 is sequestered in the cytoplasm by its negative regulator, Keap1 [41,42]. However, oxidative insults trigger the dissociation of Nrf2 from Keap1 and its subsequent translocation into the nucleus. Once in the nucleus, Nrf2 binds to the antioxidant response element (ARE) to drive the transcription of essential cytoprotective genes, including HO-1 and SOD2 [43,44,45]. These downstream effectors, such as HO-1, play a crucial role in degrading heme to generate biliverdin, carbon monoxide, and free iron, thereby inhibiting ROS accumulation and regulating immune signaling [44]. As Nrf2 serves as a core transcription factor for maintaining hepatic homeostasis [43], our study observed that rutin pretreatment significantly downregulated Keap1 while upregulating Nrf2 and its downstream effectors. Although Keap1 levels increased slightly after rutin treatment, no statistically significant difference was observed between the rutin-treated and control groups. This observation suggests that rutin exerts its biological effects by modulating the interaction between Keap1 and Nrf2, rather than altering the total abundance of Keap1. Specifically, the dissociation capacity of Nrf2 from the Nrf2/Keap1 complex might be enhanced. Importantly, the downstream effectors SOD2 and HO-1 did not exhibit significant alterations with rutin treatment alone but were markedly upregulated by rutin under H_2_O_2_-induced oxidative stress. This suggests that rutin targets the Nrf2/Keap1 signaling pathway to counteract oxidative stress. These findings align with previous studies in mammalian and avian species, where flavonoids regulate the Nrf2/Keap1 signaling pathway to induce antioxidant defense [45,46,47,48]. In avian species, the Nrf2/HO-1 signaling pathway is particularly critical for maintaining systemic homeostasis [49]. This study further confirms that rutin effectively mitigates H_2_O_2_-induced damage in primary chicken embryo hepatocytes by activating the Nrf2/Keap1 axis to upregulate HO-1 and SOD2.

The avian liver is the primary site of de novo lipogenesis, making it highly susceptible to metabolic disorders when redox signaling is disrupted. Intracellular lipid droplets, particularly triglycerides, serve as core elements in energy storage and membrane homeostasis in biological systems. Notably, rutin has been shown to inhibit lipid synthesis and accumulation while promoting metabolic homeostasis [50]. Previous studies have shown that dietary inclusion of rutin significantly reduces TG and T-CHO levels in poultry liver by inhibiting SREBP1 and downregulating its target gene expression [48]. Similarly, rutin-rich extracts can lower serum TG, T-CHO, and LDL-C concentrations, thereby improving lipid metabolism and exerting a protective effect [51,52]. Consistent with these findings, we observed that oxidative stress acts as a potent trigger for lipogenic dysregulation, evidenced by upregulation of SREBP1 and the accumulation of TG, T-CHO, and LDL-C. However, rutin still reduced T-CHO levels even under ZAKα overexpression. Previous studies have demonstrated that rutin can lower both TG and T-CHO [53,54]. This discrepancy suggests that rutin may regulate cholesterol homeostasis through pathways independent of the ZAKα-mediated ribotoxic stress response. Considering that SREBP1 primarily governs fatty acid and triglyceride synthesis, whereas cholesterol metabolism is predominantly orchestrated by SREBP2 and HMG-CoA reductase (HMGCR) [55], it is plausible that rutin directly modulates cholesterol-related enzymes or transport mechanisms that bypass the ZAKα-p38/JNK signaling axis. Beyond the ZAKα target, several pathways have been identified as potential targets for rutin, including the PI3K/AKT/mTOR [56] and AMPK pathway [53], which are widely involved in cholesterol metabolism and transport [57,58]. It is inferred that rutin may modulate these pathways to exert its cholesterol-lowering effects. These findings extend our understanding by showing that rutin’s activation of Nrf2 is not an isolated event but is closely linked to the suppression of upstream stress signals, thereby ensuring a sustained, high-capacity defense against ROS-induced lipid peroxidation and accumulation.

During oxidative stress, the disruption of ROS homeostasis and subsequent activation of kinase phosphorylation cascades are fundamental drivers [59]. Emerging evidence indicates that ROS can activate ZAKα and the downstream RSR, thereby triggering p38 and JNK cascades that induce oxidative stress [26,30]. These mechanisms provide a novel framework for understanding hepatic injury. Accumulating evidence demonstrates that rutin can effectively inhibit the p38/JNK signaling axis across various studies. One study found that rutin enhances antioxidant defenses in chicken livers by blocking p38/JNK and NF-κB signaling [21]. Similarly, rutin-rich mulberry extract downregulates the mRNA expression of p38 and JNK in human gastric mucosal epithelial cells, alleviating oxidative stress [60]. Rutin also alleviates UV-induced skin damage in mice by inhibiting the phosphorylation of p38 and JNK [61]. While these studies collectively suggest that rutin exerts antioxidant and protective effects by inhibiting p38 and JNK, the specific upstream sensors involved—particularly whether rutin regulates the Nrf2/Keap1 pathway through ZAKα-mediated phosphorylation of p38 and JNK—have not been explored until now. It is well established that p38/JNK typically modulates the Nrf2/Keap1 pathway by phosphorylating Nrf2, thereby inhibiting its nuclear translocation [62]. This mechanism is further supported by observations that nuclear Nrf2 localization decreases upon p38 activation [63]. Our study indicates that rutin significantly suppresses ribotoxic stress by inhibiting the upregulation of ZAKα and reducing p-p38/p-JNK levels, ultimately alleviating oxidative stress responses. Further experiments demonstrate that ZAKα overexpression attenuates rutin’s inhibition of p38/JNK and negates rutin’s protective role against H_2_O_2_-induced oxidative stress and lipid peroxidation. These findings suggest that rutin exerts its regulatory function via ZAKα, potentially through a direct interaction with ZAKα. Given that ZAKα upregulation is known to reduce survival and induce apoptosis in human osteosarcoma cells [64], our findings highlight ZAKα as a potential target for nutritional intervention. AlphaFold-based molecular docking, frequently used to elucidate potential intermolecular interactions [65], shows a binding energy of −8.6 kcal/mol between rutin and ZAKα, confirming their binding relationship. The involvement of specific residues suggests that rutin may interfere with the structural flexibility or ATP-binding capacity of ZAKα, thereby suppressing its kinase activity. This structural interaction provides a plausible explanation for our observation that rutin effectively dampens activation of the downstream p38/JNK signaling cascade. ZAKα is recognized as a key sensor of ribotoxic stress [26], capable of translating ribosomal stalling into oxidative stress signals. Our results support the hypothesis that rutin acts as a direct molecular antagonist of ZAKα, thereby preserving the Nrf2-mediated antioxidant response. Although ZAKα was identified as a target, a limitation of this study is that the in vitro hepatocyte model does not fully capture the inter-organ crosstalk present in vivo. Future research integrating in vivo feeding trials and multi-omics approaches is required to bridge the gap between these mechanistic findings and practical applications in poultry nutrition.

## 5. Conclusions

**Figure 10:**
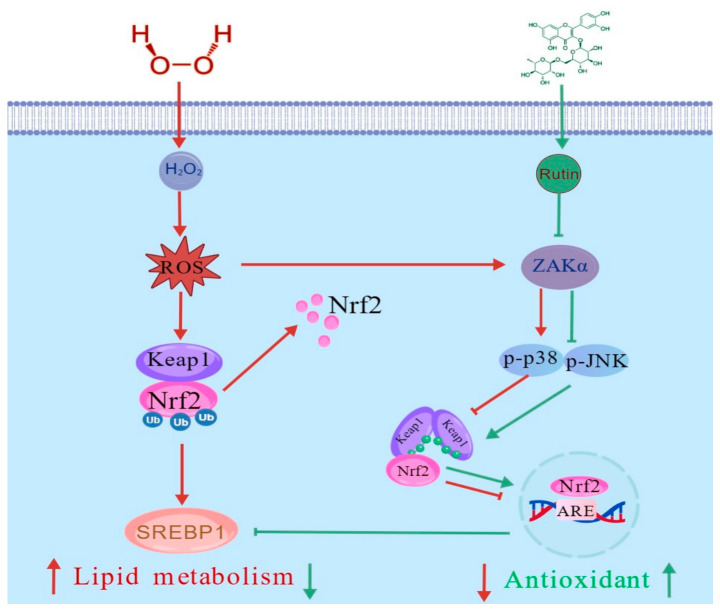
Schematic diagram illustrating the mechanism by which rutin alleviates oxidative stress in chicken liver cells by inhibiting the nuclear toxicity stress response. Rutin enhances antioxidant capacity by activating the Nrf2/Keap1 pathway while inhibiting ZAKα-mediated p38/JNK inflammatory signaling, thereby alleviating oxidative stress and improving lipid metabolism.

In summary, our study demonstrates that rutin alleviates oxidative injury and lipid accumulation in chicken embryo hepatocytes by modulating two interconnected signaling pathways. Rutin activates the Nrf2/Keap1 pathway to enhance antioxidant defenses and downregulates SREBP1 to inhibit abnormal lipid accumulation. Importantly, we identified the ZAKα-p38/JNK-mediated ribotoxic stress response as a critical upstream target of rutin. Overexpression of ZAKα effectively counteracts rutin’s protective functions, confirming that rutin’s efficacy is mechanistically dependent on the suppression of ZAKα signaling (Figure 10). These findings establish ZAKα as a novel target for nutritional interventions and provide a theoretical foundation for using rutin as a functional additive to improve poultry liver health. However, the mechanism has been validated only in an in vitro model and has not yet been confirmed in vivo. The systemic efficacy and tissue-specific regulatory networks of the ZAKα axis require validation in whole-animal poultry models.

## Data Availability

Data will be made available on request.
